# Hantavirus Brno loanvirus is highly specific to the common noctule bat (*Nyctalus noctula*) and widespread in Central Europe

**DOI:** 10.1007/s11262-022-01952-2

**Published:** 2022-12-21

**Authors:** Maysaa Dafalla, Anna Orłowska, Sinan Julian Keleş, Petra Straková, Kore Schlottau, Kathrin Jeske, Bernd Hoffmann, Gudrun Wibbelt, Marcin Smreczak, Thomas Müller, Conrad Martin Freuling, Xuejing Wang, Jerzy Rola, Stephan Drewes, Sasan Fereidouni, Gerald Heckel, Rainer G. Ulrich

**Affiliations:** 1 Institute of Novel and Emerging Infectious Diseases, Friedrich-Loeffler-Institut, Federal Research Institute for Animal Health, Südufer 10, 17493 Greifswald-Insel Riems, Germany; 2 Department of Virology, National Veterinary Research Institute, 57 Partyzantów Avenue, 24-100 Pulawy, Poland; 3 Research Institute of Wildlife Ecology, University of Veterinary Medicine Vienna, Savoyenstraße 1a, 1160 Vienna, Austria; 4 Veterinary Research Institute, Hudcova 296/70, 621 00 Brno, Czech Republic; 5 Institute of Diagnostic Virology, Friedrich-Loeffler-Institut, Federal Research Institute for Animal Health, Südufer 10, 17493 Greifswald-Insel Riems, Germany; 6 Leibniz Institute for Zoo and Wildlife Research, Alfred-Kowalke-Straße 17, 10315 Berlin, Germany; 7 Institute of Molecular Virology and Cell Biology, Friedrich-Loeffler-Institut, Federal Research Institute for Animal Health, Südufer 10, 17493 Greifswald-Insel Riems, Germany; 8 Institute of Ecology and Evolution, University of Bern, Baltzerstrasse 6, 3012 Bern, Switzerland; 9 Quartier Sorge - Batiment Amphipole, Swiss Institute of Bioinformatics, 1015 Lausanne, Switzerland; 10 German Center for Infection Research (DZIF), Partner Site Hamburg-Lübeck-Borstel-Riems, Südufer 10, 17493 Greifswald-Insel Riems, Germany

**Keywords:** Hantavirus, Bats, RT-qPCR, Host, Europe, Host specificity

## Abstract

Bat-associated hantaviruses have been detected in Asia, Africa and Europe. Recently, a novel hantavirus (Brno loanvirus, BRNV) was identified in common noctule bats (*Nyctalus noctula*) in the Czech Republic, but nothing is known about its geographical range and prevalence. The objective of this study was to evaluate the distribution and host specificity of BRNV by testing bats from neighbouring countries Germany, Austria and Poland. One thousand forty-seven bats representing 21 species from Germany, 464 bats representing 18 species from Austria and 77 bats representing 12 species from Poland were screened by L segment broad-spectrum nested reverse transcription-polymerase chain reaction (RT-PCR) or by BRNV-specific real-time RT-PCR. Three common noctules from Germany, one common noctule from Austria and three common noctules from Poland were positive in the hantavirus RNA screening. Conventional RT-PCR and primer walking resulted in the amplification of partial L segment and (almost) complete S and M segment coding sequences for samples from Germany and partial L segment sequences for samples from Poland. Phylogenetic analysis of these nucleotide sequences showed highest similarity to BRNV from Czech Republic. The exclusive detection of BRNV in common noctules from different countries suggests high host specificity. The RNA detection rate in common noctules ranged between 1 of 207 (0.5%; Austria), 3 of 245 (1.2%; Germany) and 3 of 20 (15%; Poland). In conclusion, this study demonstrates a broader distribution of BRNV in common noctules in Central Europe, but at low to moderate prevalence. Additional studies are needed to prove the zoonotic potential of this hantavirus and evaluate its transmission within bat populations.

**Supplementary Information:**

The online version contains supplementary material available at 10.1007/s11262-022-01952-2.

Hantaviruses, family *Hantaviridae*, order *Bunyavirales*, are currently classified into four genera: *Orthohantavirus*, *Thottimvirus*, *Mobatvirus* and *Loanvirus* [1, 2]. The hantavirus genome consists of three RNA segments of negative polarity. The small (S) segment encodes the nucleocapsid (N) protein and the large (L) segment encodes the RNA-dependent RNA polymerase (RdRp). The glycoprotein precursor (GPC) is encoded by the medium (M) segment and cotranslationally cleaved at a conserved sequence motif into N-terminal Gn and C-terminal Gc [3].

Hantavirus disease in humans is currently thought to be associated with certain rodent-borne hantaviruses. Haemorrhagic fever with renal syndrome is caused by different hantaviruses in the Old World and by Seoul orthohantavirus, detected in both the Old and New World. In the New World, hantavirus cardiopulmonary syndrome is caused by Andes orthohantavirus, Sin nombre orthohantavirus [4], and related viruses.

Most rodent-borne hantaviruses exhibit strong host specificity, explained by virus–host coevolution [5, 6]. The host association of hantaviruses is usually evaluated by field studies of sympatric small mammals. Here, the reservoir is defined by the most frequent molecular detection of a given hantavirus in a single species or closely related species [6]. The geographical distribution of a given hantavirus follows the range of its reservoir; however, phylogeographic processes and ecological factors influencing reservoir populations may result in the absence of a hantavirus in certain areas [7–9].

Hantaviruses were initially thought to be exclusively rodent-borne, despite the first hantavirus being isolated from the Asian house shrew, *Suncus murinus* [10]. Moreover, the use of broad-spectrum L segment reverse transcription-polymerase chain reaction (RT-PCR) assays resulted in the identification of novel hantaviruses in shrews and moles [11–22]. Furthermore, novel hantaviruses have also recently been detected and characterized in bat species from Africa (e.g. Mouyassue virus (MOYV) in Cote d'Ivoire; Magboi virus (MGBV) in Sierra Leone; Makokou virus (MAKV) in Gabon) and Asia (e.g. Xuan Son virus (XSV) in Vietnam; Huangpi virus (HUPV), Longquan loanvirus (LQUV); Laibin mobatvirus (LBV) in China; Quezon mobatvirus (QZNV) in the Philippines; Đakrông virus (DKGV) in Vietnam) [23–30]. For most of these viruses, the host specificity is not well documented. The recent finding of Brno loanvirus (BRNV) in common noctule bats (*Nyctalus noctula*) shows the presence of bat-borne hantaviruses in Europe [31].

Here we aimed to evaluate the presence, prevalence and host specificity of BRNV through a field survey of bats from Germany, Austria and Poland.

Bats and/or bat swab samples were collected from Poland, Austria and Germany [32] (Fig. 1a). Animal carcasses were dissected, and tissue samples and body cavity fluid were collected following standard protocols. Bat species were determined by morphological characters or molecular methods (for details see Supplementary Information). Tissue as well as oropharyngeal and rectal swab samples were subjected to RNA isolation and conventional RT-PCR and a novel BRNV-specific real-time RT-qPCR both targeting the L segment (for primer sequences and binding site positions see Table S1). Phylogenetic and sliding window analyses of sequence data and prediction of functional domains followed standard procedures (see Supplementary Information).

**Fig. 1:**
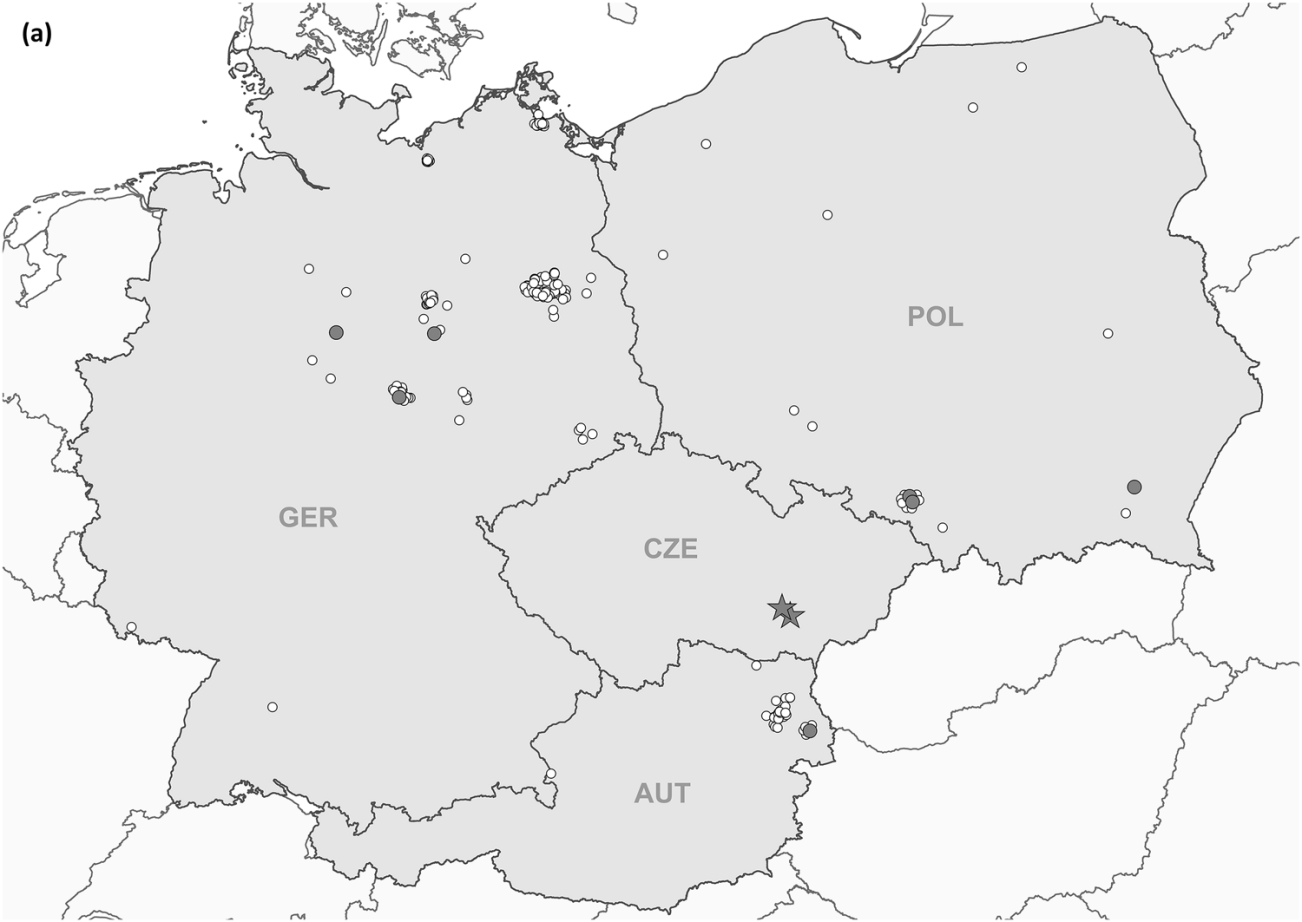

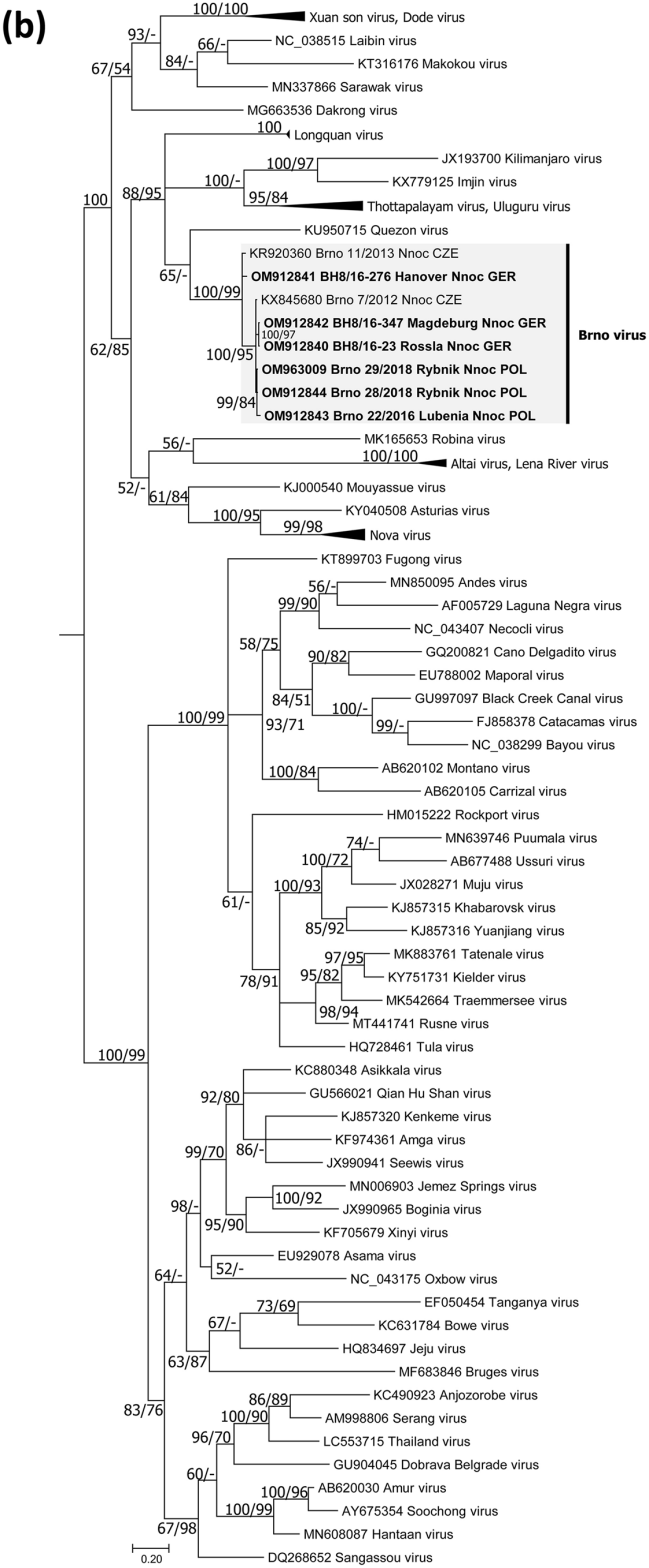

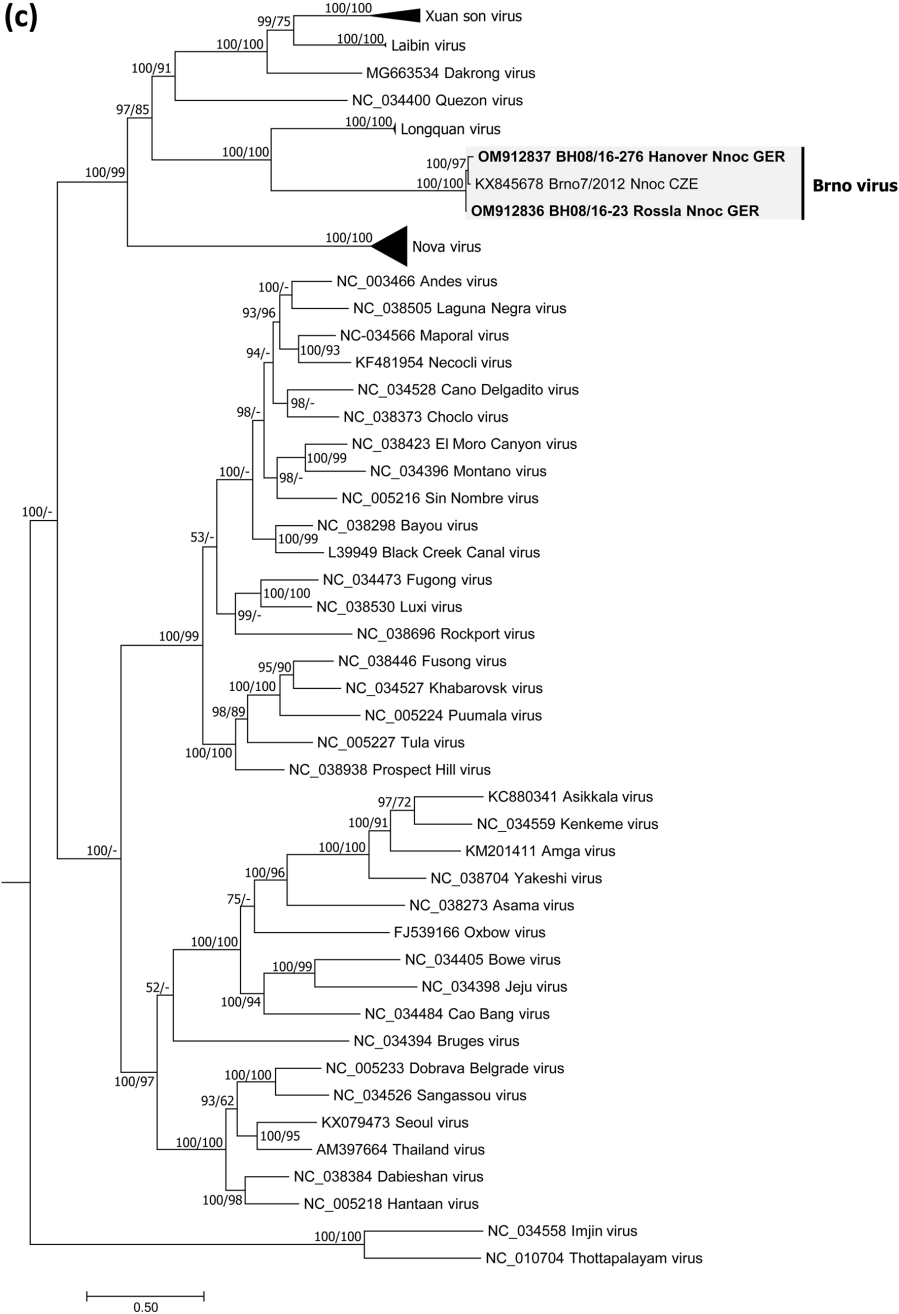

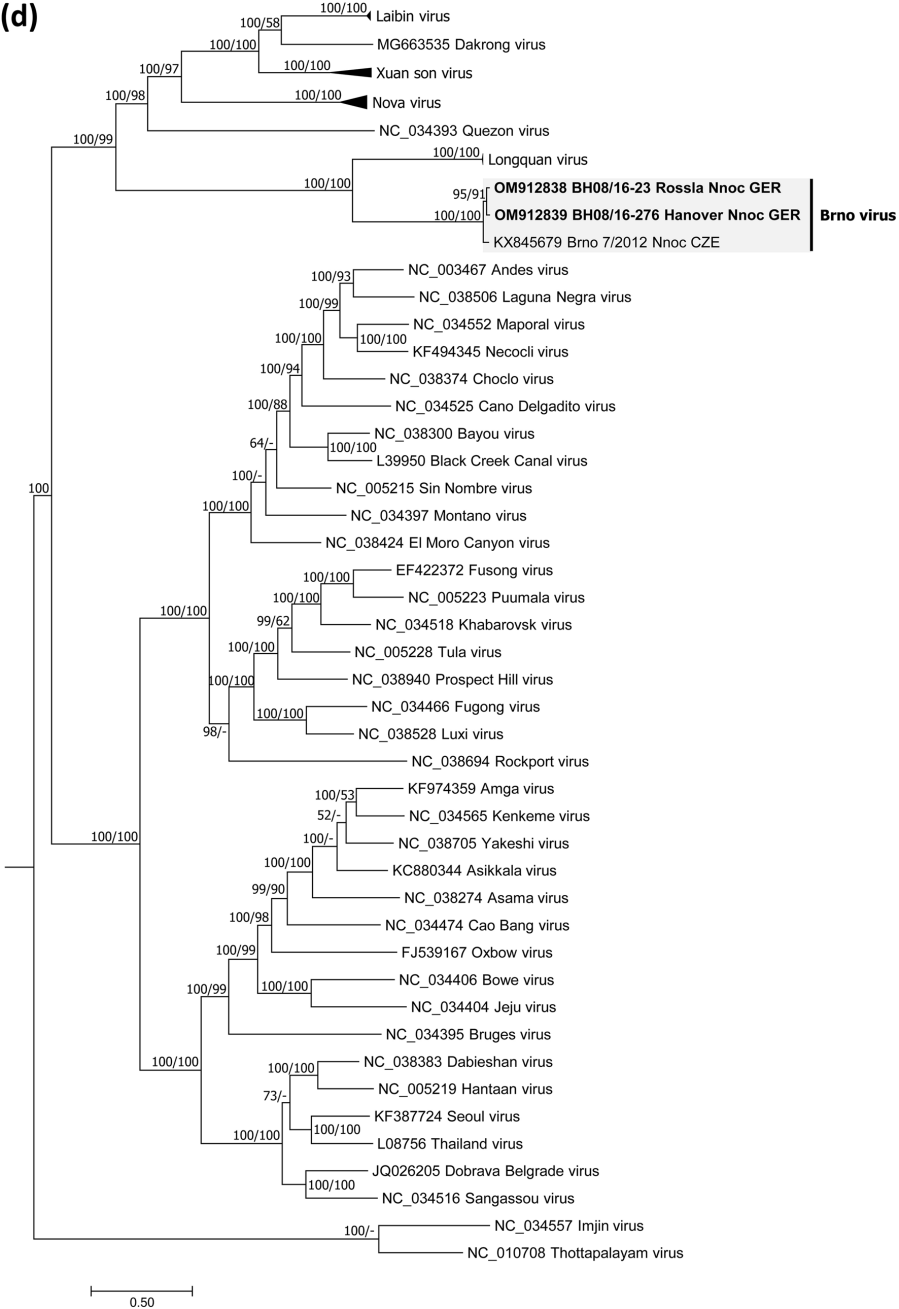
Geographical origin of bat samples and evolutionary relationships of Brno loanvirus (BRNV) sequences. **a** Geographical origin of common noctule bat (*Nyctalus noctula*) samples from Germany (*n =* 245, GER), Austria (*n =* 207, AUT) and Poland (*n =* 20, POL) tested for BRNV. The geographical origin of the two noctule bats from the Czech Republic (city of Brno, CZE) where BRNV was discovered is indicated. The origin of BRNV RNA positive and negative common noctules is indicated in the map by black dots and grey dots, respectively. For clarity, the sampling locations of BRNV-negative bats (*N =* 1133) from 20 other species are not shown. **b–d** Phylogenetic consensus trees of partial L segment (**b**), (almost) complete S segment (**c**) and M segment (**d**) sequences of the novel BRNV strains, prototype BRNV strain from the Czech Republic, Longquan loanvirus and reference sequences of the other *Mammantavirinae* genera. Alignments were constructed using the Clustal W Multiple Alignment algorithm implemented in Bioedit (V7.2.3.) [38]. The most suitable substitution model was determined by jModelTest v2.1.6 [39]. The consensus trees are based on Bayesian analyses with 6 × 10^6^ to 1 × 10^7^ generations and a burn-in phase of 25% using MrBayes v3.2.6 [40] and Maximum-Likelihood analyses with 1000 bootstrap replicates performed with the aid of FasttreeMP v2.1.10 [41] and 50% cut-off using the General Time Reversible (GTR) substitution model with invariant sites and a gamma-distributed shape parameter for both algorithms. The consensus tree was established by transferring the bootstrap values to the Bayesian tree only if branches were supported by both trees. All tree reconstructions were done on CIPRES Science Gateway [42]. *Nnoc* Nyctalus noctula

Screening of organ pool samples, from 1047 bats from 21 species from Germany [33], by RT-qPCR resulted in the identification of three positive samples (Table 1). Of the 618 oropharyngeal and rectal swab samples and 155 pooled tissue samples collected from 464 bats, representing 18 species, from Austria one tissue sample was positive (Table 1). Additional screening of the 1047 bats from Germany by conventional RT-PCR tested the same three samples positive and therefore confirmed the results of the RT-qPCR analysis (see Table 1). Subsequent analyses of common noctules (*N* = 245) from Germany by RT-PCR assays targeting partial M and S segment revealed two positive samples each; in contrast, re-amplification of the RT-qPCR-positive sample from Austria by conventional RT-PCR failed, most likely due to a lower sensitivity of the conventional assay. Of the 77 bats belonging to 12 species from Poland, three common noctules were positive by conventional nested RT-PCR of lung, liver and kidney samples (Table 1). All positive samples originated from common noctules with an estimated prevalence of 1.2% (3 of 245; 95% confidence interval, CI, 0.25–3.5%) for Germany, 0.5% (1 of 207; CI 0.01–2.7%) for Austria and 15% (3 of 20; CI 3.2–37.9%) for Poland. The here observed prevalence for common noctules in Poland might be biased by the low number of samples. The positive common noctules originated from two sites in central Germany, one site in north-western Germany, one site in Lower Austria and two locations in Southern Poland (Fig. 1a).

**Table 1:** Results of Brno loanvirus-specific RT-qPCR screening of bats from Germany and Austria and of nested RT-PCR for samples from Poland

Family	Subfamily	Genus	Common name	Scientific name	No. of positive animals/total no. of animals		
					Germany*	Austria	Poland
Vespertilionidae	Vespertilioninae	*Nyctalus*	Common noctule	*Nyctalus noctula*	3/245	1/207	3/20
			Lesser noctule	*Nyctalus leisleri*	0/19	0/1	–
		*Pipistrellus*	Common pipistrelle	*Pipistrellus pipistrellus*	0/387	0/30	0/5
			Soprano pipistrelle	*Pipistrellus pygmaeus*	0/6	0/3	0/4
			Nathusius' pipistrelle	*Pipistrellus nathusii*	0/51	0/33	0/3
			Kuhl's pipistrelle	*Pipistrellus kuhlii*	0/3	0/45	–
			Savi's pipistrelle	*Hypsugo/Pipistrellus savii*	–	0/51	–
		*Vespertilio*	Parti-coloured bat	*Vespertilio murinus*	0/23	0/43	0/5
		*Plecotus*	Brown long-eared bat	*Plecotus auritus*	0/43	–	0/4
			Grey long-eared bat	*Plecotus austriacus*	0/16	0/11	–
		*Eptesicus*	Northern bat	*Eptesicus nilssonii*	0/3	–	0/2
			Serotine bat	*Eptesicus serotinus*	0/56	0/7	0/17
		*Barbastella*	Western barbastellus	*Barbastella barbastellus*	0/4	0/11	–
	Myotinae	*Myotis*	Bechstein's bat	*Myotis bechsteinii*	0/6	–	–
			Brandt's bat	*Myotis brandti*	0/16	–	–
			Pond bat	*Myotis dasycneme*	0/3	–	0/1
			Daubenton's bat	*Myotis daubentonii*	0/46	0/2	0/3
			Greater mouse-eared bat	*Myotis myotis*	0/18	0/1	–
			Whiskered bat	*Myotis mystacinus*	0/48	0/6	0/2
			Natterer's bat	*Myotis nattereri*	0/47	–	–
			Alcathoe Whiskered bat	*Myotis alcathoe*	0/2	–	0/1
			Geoffroy's bat	*Myotis emarginatus*	–	0/2	–
Pteropodidae	Pteropodinae	*Rousettus*	Egyptian fruit bat	*Rousettus aegyptiacus*	0/2	–	–
Miniopteridae	Miniopterinae	*Miniopterus*	Schreiber's bent-winged bat	*Miniopterus schreibersii*	–	0/5	–
Rhinolophidae	Rhinolophinae	*Rhinolophus*	Greater horseshoe bat	*Rhinolophus ferrumequinum*	–	0/3	–
			Lesser horseshoe bat	*Rhinolophus hipposideros*	–	0/2	–
Species not identified**					0/3	0/1	0/10
Total					3/1047	1/464	3/77

^*^Results of the RT-qPCR were confirmed by conventional RT-PCR, detecting the same three samples as positive

^**^for three samples from Germany, one sample from Austria and 10 samples from Poland the bat species could not be identified

Phylogenetic analysis confirmed high similarity between common noctule-derived partial L segment sequences from this study and two recently discovered sequences from the Czech Republic [31], but showed a clear divergence to other bat-, insectivore- and rodent-borne hantaviruses (Fig. 1b). The nucleotide and amino acid sequence identity of the partial L segment and RdRp sequences of BRNV strains ranged between 94.3–100% and 95.2–100%, respectively (Table S2).

A primer-walking-based approach (for details see Supplementary Information) resulted in the determination of the almost complete coding sequences (CDS) of the S and M segments of BRNV from two common noctules from Germany (sample BH 08/16–276 / sample BH 08/16–23). Phylogenetic analyses and pairwise sequence comparisons of the CDS confirmed the high similarity to the BRNV prototype sequences from the Czech Republic (Fig. 1c, d; Table S3). The BRNV sequences of both segments were clearly separated from sequences of other bat-borne hantaviruses and the rodent- and insectivore-borne hantaviruses.

The N protein of BRNV prototype strain CZE 7_2012 of 423 amino acid residues was predicted to contain a non-cytoplasmic domain, a transmembrane domain, a cytoplasmic domain and three coiled coil structures (Fig. S1a). Amino acid sequence diversity of the novel BRNV sequences was highest within the transmembrane domain and at the N terminus of the non-cytoplasmic domain/coiled coil structures.

The prototype strain GPC (1136 residues) was predicted to consist of an 18 amino acid signal peptide at the N terminus of Gn, followed by a non-cytoplasmatic domain, a transmembrane domain and a cytosolic tail, including an immunoreceptor tyrosine-based activation (ITAM) motif, followed by the putative Gn/Gc pentapeptide cleavage site (residues 649–653; Fig. S1b). This pentapeptide in the prototype and the novel BRNV strains (“W**GS**SA”) differ from the conserved motif “WAASA” found in most orthohantaviruses [34, 35], mobatviruses and all thottimviruses, but also from the putative cleavage site “WA**S**SA” in Longquan loanvirus and “WA**V**SA” in Asama, Bruges and Fugong orthohantaviruses (Table S4). The predicted Gc protein includes a non-cytoplasmatic domain and ends after a transmembrane domain in a short cytoplasmic tail. The highest sequence divergence was seen within the Gn part of GPC, with peaks at the N terminus and the cytoplasmic domain (see Fig. S1b).

The tissue distribution of BRNV RNA was investigated for the three common noctules from Germany by RT-qPCR analyses. The highest viral RNA loads were observed in liver, lung and kidney samples (Table S5). In one animal, the brain and spleen samples were positive, whilst in another, the intestine sample was positive. The conventional RT-PCR analyses of different tissues of the three bats from Poland revealed the detection of viral RNA in the livers of two bats and in one kidney and one lung sample (Table S5).

This study detected BRNV in common noctules from Germany, Poland and Austria, suggesting a broader geographical distribution of this hantavirus in Central Europe. The multiple detection of this hantavirus in the same bat species from different regions, together with the absence of BRNV-specific RNA in other sympatric bat species, indicated its clear host specificity to the common noctule.

It should be noted that the prevalence estimates in common noctules from Germany, Austria (this study) and the Czech Republic [31] might be affected by inherent biases in sampling methods. For the prevalence in common noctules from Poland, it is also noteworthy that two BRNV-positive bats were found dead at the same place and time in Silesia. The overall intra-species sequence divergence was found to be rather low, both at the nucleotide sequence level, for S, M and L segments (max. 2.3%, 4.4%, and 6.4%), and amino acid levels, for the encoded proteins (0%, 0.7%, 2.9%), in comparison to the intra-species sequence divergence in rodent-borne hantaviruses.

The common noctule (*Nyctalus noctula*) is one of the largest bats in Western and Central Europe and common throughout Europe, Asia, and North Africa [36]. Common noctules typically live in forests, but populations can also be found in human settlements [37]. The occurrence of this bat species in urban regions may raise a public health concern due to a potential risk of transmission of this bat-borne hantavirus to humans. The characterization of BRNV, its host association and geographical distribution may ultimately assist in the prevention of its emergence in humans and other animals. Future investigations in other regions where common noctules are endemic will profit from our novel RT-qPCR assay, which shows here an (almost) identical sensitivity to the nested RT-PCR assay for BRNV detection, at least in Germany. The performance of this assay should be validated in the future based on synthetic RNA molecules, as long as a cell culture isolate of BRNV is lacking. Further studies will be dedicated to analyse the zoonotic potential of this bat-borne hantavirus and evaluate its transmission within bat populations.

## Supplementary Information

Below is the link to the electronic supplementary material.

Supplementary file1 (DOCX 205 kb)

Supplementary file2 (DOCX 45 kb)

Supplementary file3 (DOCX 39 kb)

## Data Availability

The entire information on the bats investigated here is given within the manuscript and the Supplementary Information. All new Brno loanvirus sequences are deposited at GenBank under the accession numbers OM912836- OM912844.
