# Supplementary material for: Hantavirus Brno loanvirus is highly specific to the common noctule bat (Nyctalus noctula) and widespread in Central Europe
Source: Virus Genes. 2022 Dec 21;59(2):323–32. doi: 10.1007/s11262-022-01952-2 (PMC10025241; doi:10.1007/s11262-022-01952-2)
Supplement: Supplementary file 1 — Supplementary file1 (DOCX 205 kb) [file 11262_2022_1952_MOESM1_ESM.docx]

Figure S1. Sliding-window analyses of amino acid diversity along the nucleocapsid protein (a) and glycoprotein precursor (b) within each hantavirus clade, and amino acid divergence between clade I and clade IIa or clade IIb (see Figure 1c, d). Windows of 40 amino acids were shifted in steps of 20 amino acids. Functional domains of the proteins were predicted for the Brno loanvirus prototype strain Brno 7/2012 Nnoc (GenBank accession numbers KX845678 and KX845679) using the InterPro Classification of protein families from the European Molecular Biology Laboratory, European Bioinformatic Institute (EMBL-EBI (<https://www.ebi.ac.uk/interpro/>). Hantavirus clade assignments were used according to Figure 1, b-d: Clade I - Brno (Hanover Nnoc BH08_16_276), Longquan (NC_043126), Laibin (NC_038514), Nova (NC_034464), Quezon (NC_034400), Xuan son (KY662273), Đakrông (MG663534); Clade IIa - Asama (NC_038273), Asikkala (KC880341), Bruges (NC_034394), Hantaan (NC_005218), Jeju (NC_034398), Dobrava Belgrade (NC_005233), Yakeshi (NC_038704); Clade IIb - Bayou (NC_038298), Cano Delgadito (NC_034528), Sin Nombre (NC_005216), Fugong (NC_034473), Puumala (NC_005224), Andes (NC_003466), Tula (NC_005227).
