# Supplementary material for: Hantavirus Brno loanvirus is highly specific to the common noctule bat (Nyctalus noctula) and widespread in Central Europe
Source: Virus Genes. 2022 Dec 21;59(2):323–32. doi: 10.1007/s11262-022-01952-2 (PMC10025241; doi:10.1007/s11262-022-01952-2)
Supplement: Supplementary file 2 — Supplementary file2 (DOCX 45 kb) [file 11262_2022_1952_MOESM2_ESM.docx]

**Table S1: Primers and probe used for real-time and conventional RT-PCR amplification of S, M and L segment sequences**

| Segment/PCR | Name | Sequence (5´ - 3´) | References |
| --- | --- | --- | --- |
| Screening real-time RT-PCR L segment | | | |
|  | Hanta-Brno-3152-F | GAT TAG TTA GAG GTA ATT GGT TAC A | This study |
|  | Hanta-Brno-3237-R | TCM GAG AAT AAT TCA CTC CAA ACT | This study |
|  | Hanta-Brno-3192-FAM | FAM-ATG TTC ATC AYT ATT TAG CGT TGC AGT TAG-BHQ1 | This study |
| Partial sequences | | | |
| S segment | Hanta_S_280-F | CGG TAC GGA AAT GTT ATT GAT TT | This study |
|  | Hanta_S_914-R | GKG GRC AYC TAT CAG GTG | This study |
| M segment | Hanta_M_118_fw | CATG GCA GCG TAG ATT TAG ACC | This study |
|  | Hanta_M_1083_rev | AGT CCC TGT CCA TAC TAG TGG T | This study |
| L segment | Han-L-F1_2896 | ATG TAY GTB AGT GCW GAT GC | [[1](#_ENREF_1)] |
|  | Han-L-R1_3334 | AAC CAD TCW GTY CCR TCA TC | [[1](#_ENREF_1)] |
|  | Han-L-F2_2907 | TGC WGA TGC HAC IAA RTG GTC | [[1](#_ENREF_1)] |
|  | Han-L-R2_3281 | GCR TCR TCW GAR TGR TGD GCA A | [[1](#_ENREF_1)] |
|  | Hanta_L_2821-F | GCA TTR AGA TGG GCT TCA | This study |
|  | Hanta_L_3586-R | AAR AAA CCT AAM CCT GGT AAA TC | This study |
|  |  |  |  |
| Complete sequences | | | |
| S segment | BrnoV-N-256-F | CAT TTA ACT GAA CGT TCA WCA YTA C | This study |
|  | BrnoV-N-282-F | GTA CGG RAA TGT TAT TGA TYT GGA | This study |
|  | BrnoV-N-679-R | TGT CAA TAT GTT CTY CCC AWT TTT C | This study |
|  | BrnoV-N-906-R | CAT CKA TCA GGT GAA CCT GCA | This study |
|  | BrnoV-N-642-F | ACC TGT GAT GGG WGT WAT TGG A | This study |
|  | BrnoV-N-1153-R | GAG RTG GAA ATG ATC AAC CAT CAT | This study |
|  | BrnoV-N-1180-R | CTK AGC TCA GMG TCC ATA TCA TC | This study |
|  | BrnoV-N-43-F | GAA AGA GAA ATT ACT AAA GCC AAG C | This study |
|  | BrnoV-N-326-R | ATC TGC TGT CCC TCC ACT AG | This study |

**Table S1 (continued)**

| Segment/PCR | Name | Sequence (5´ - 3´) | References |
| --- | --- | --- | --- |
| M segment | BrnoV-G-10-F | CTC CAC GAA AAT GAA GTC AMT ACT | This study |
|  | BrnoV-G-725-R | GAC ATA ACC MCC WGA GTA TTC AG | This study |
|  | BrnoV-G-1318-F | TGT AAT GGT GAA AAY ATM ACA GTT CC | This study |
|  | BrnoV-G-1981-R | ATA CCR TGA GCT GTA TYT TGC CA | This study |
|  | BrnoV-G-2474-F | ATG AYT GTT TGG TWT CAA ATG GTG T | This study |
|  | BrnoV-G-2612-F | GTA ART TTG GTG ATC CTG GWG AT | This study |
|  | BrnoV-G-3114-R | CAG AGC CAC TAT GAC CYC CT | This study |
|  | BrnoV-G-1844-F | CYT ATT CAA GAA GAG TCA GAC G | This study |
|  | BrnoV-G-1887-F | AAA TAG ATG YTA TAT AAT GAC AAT ATG G | This study |
|  | BrnoV-G-2557-R | ACC ACC TAC TAA AGG TCC AAG | This study |
|  | BrnoV-G-2596-R | AAA CTT ACA ATT TGA TGT GCA CCA | This study |
|  | BrnoV-G-2248-R | GGC ATG CGG CTA ATT GCC A | This study |
|  | BrnoV-G-2278-R | CCA ATT ACT TTG AAA TGC ATA GTC | This study |
|  | BrnoV-G-2172-F | TAA TTG GAT GGA TGG AGA AGT CA | This study |
|  | BrnoV-G-2198-F | TAA AAT CAG TTT TCC ACT GCT GG | This study |

Primer position numbers according to reference strains KX845678 Brno 7 2012 Nnoc CZE, KX845679 Brno 7 2012 Nnoc CZE and KX845680 Brno 7 2012 Nnoc CZE for S, M and L segment, respectively.

**Table S2: Pairwise L segment nucleotide (nt) and amino acid (aa) sequence identity (in %) of partial L segment and RNA-dependent RNA polymerase, respectively, of the novel strains from Germany (DE) and Poland (POL), the prototype strain of Brno loanvirus and other related hantaviruses**

| Reference sequences | BH08/16-23  Rossla_Nnoc_GER | | BH08/16-276 Hanover_Nnoc_GER | | BH08/16-347 Magdeburg_Nnoc_GER | | Brno_22_2016  POL | | Brno_28_2018  POL | |
| --- | --- | --- | --- | --- | --- | --- | --- | --- | --- | --- |
|  | nt | aa | nt | aa | nt | aa | nt | aa | nt | aa |
| BH_8_16_276_GER | 94.3 | 99 | ID | ID | 94.3 | 97.1 | 96.2 | 98 | 95.2 | 98 |
| BH_8_16_347_GER | 99.3 | 98 | 94.3 | 97.1 | ID | ID | 96.5 | 95.2 | 97.7 | 97.1 |
| BH_8_16_23_GER | ID | ID | 94.3 | 99 | 99.3 | 98 | 97.1 | 97.1 | 98.4 | 99 |
| Brno_22_2016_POL | 97.1 | 97.1 | 95.2 | 98 | 96.5 | 95.2 | ID | ID | 98.7 | 98 |
| Brno_28_2018_POL | 98.4 | 99 | 95.2 | 98 | 97.7 | 97.1 | 98.7 | 98 | ID | ID |
| Brno_29_2018_POL | 98.4 | 99 | 95.2 | 98 | 97.7 | 97.1 | 98.7 | 98 | 100 | 100 |
| Brno_7_2012_Nnoc_CZE: KX845680 | 98.4 | 100 | 95.2 | 99 | 97.7 | 98 | 94.6 | 97.1 | 94.6 | 99 |
| Brno_11_2013_Nnoc_CZE: KR920360 | 93.6 | 100 | 97.7 | 99 | 93.6 | 98 | 94.6 | 97.1 | 94.6 | 99 |
| Quezon_virus: NC_034401 | 69 | 75.2 | 70.6 | 75.2 | 69 | 74.2 | 68.7 | 73.3 | 69.4 | 74.2 |
| Longquan_virus: NC_043125, JX465383, JX465381, JX465382 | 76.8 | 79.5 | 76.6 | 80 | 76.5 | 78.8 | 77.7 | 78 | 77.3 | 78.5 |
| Thottapalayam_virus: KJ420573, EU001330, HQ831374, MT225399 | 70.3 | 78.5 | 71.3 | 78.5 | 70.9 | 77.6 | 71 | 76.6 | 71.8 | 77.6 |
| Uluguru_virus: JX193697 | 72.5 | 75.2 | 71.9 | 75.2 | 72.2 | 74.2 | 72.5 | 75.2 | 72.8 | 74.2 |
| Imjin_virus: KX779125 | 71.2 | 78 | 72.5 | 78 | 70.9 | 77.1 | 72.2 | 76.1 | 72.2 | 77.1 |
| Kilimanjaro_virus: JX193700 | 70.6 | 69.5 | 70.9 | 69.5 | 70 | 68.5 | 71.9 | 69.5 | 71.2 | 68.5 |

ID, identical; GER, Germany; POL, Poland; CZE, Czech Republic; Nnoc, *Nyctalus noctula*

**Table S3: Pairwise sequence similarity (in %) of (almost) complete coding nucleotide (nt) sequences of S and M segments and deduced amino acid (aa) sequences of the nucleocapsid protein (N) and glycoprotein precursor (GPC) of novel BRNV strains from Germany, prototype Brno loanvirus strain and related hantaviruses**

|  | BH08/16_23 | | | | BH08/16_276 | | | |
| --- | --- | --- | --- | --- | --- | --- | --- | --- |
| Reference sequences | S segment | | M segment | | S segment | | M segment | |
|  | nt | aa | nt | aa | nt | aa | nt | aa |
| Brno virus: BH08_16_276_Nnoc_GER | 97.7 | 100 | 97.7 | 99.5 | ID | ID | ID | ID |
| Brno virus: KX845678_7_2012_Nnoc_CZE | 98.7 | 100 | n.a. | n.a. | 97.8 | 100 | n.a. | n.a. |
| Brno virus: KX845679_7_2012_Nnoc_CZE | n.a. | n.a. | 95.6 | 99.3 | n.a. | n.a. | 95.6 | 99.5 |
| Longquan_virus: JX465417, JX465416. JX465414, JX465415, NC_043126(S), JX465400, JX465399, JX465398, JX465397, JX465402(M) | 66.7 | 65.2 | 66.3 | 62.6 | 65.9 | 65.7 | 66.3 | 62.5 |
| Laibin_virus: NC_038514, KY662264(S), NC_038513, KY662265, MK393933, MM4378M18, MM4377M17(M)_ | 58.3 | 54.6 | 55.4 | 45.1 | 58.2 | 54.6 | 54.7 | 45.1 |
| Quezon_virus: NC_034400_ NC_034393, NC_034401 | 57.8 | 52.8 | 54.6 | 44.5 | 57.3 | 52.8 | 54.6 | 44.5 |
| Xuan_son_virus: KY662273, MK393929, KF704711, KF704710, KF704709, KC688335, KY66227190(S), MK393930, KU976427, KY662272, KY662268(M)_ | 57.4 | 52.5 | 54.5 | 44.8 | 57.2 | 52.5 | 54.5. | 44.8. |
| Dakrong_virus: MG663534, VN2913B72, MG663535, MG663536 | 58.1 | 54.3 | 55.1 | 45.4 | 57.8 | 54.3 | 55.1 | 45.4 |
| Nova_virus: NC_034464, KT004445, KY780086, KX512422, KX512425, KX512418, KX512396, KX512409, KX512400, KX512403, KX512402, KX512386, KX512415, KX512372, KX512350, KX512349, KX512341(S), NC_034470, KT004446, KX512430, KX512427, KY780087, KX512428, KX512406(M) | 56.8 | 51.5 | 53.5 | 43.8 | 56.6 | 51.2 | 53.6 | 43.8 |

ID, identical; n.a., not available; GER, Germany; CZE, Czech Republic

**Table S4: Amino acid sequences of the putative Gn/Gc cleavage site in GPC of Brno loanvirus and other representative strains of the genera *Loanvirus*, *Mobatvirus*, *Orthohantavirus* and *Thottimvirus*.**

| Genus | Species | Host | „WAASA“-motif | Amino acid position |
| --- | --- | --- | --- | --- |
| *Loanvirus* | Brno | Bat | W**GS**SA | 649-653 |
|  | Longquan | Bat | WA**S**SA | 646-650 |
| *Mobatvirus* | Laibin | Bat | WAASA | 641-645 |
|  | Nova | Mole | WAASA | 641-645 |
|  | Quezon | Bat | WA**V**SA | 645-649 |
| *Orthohantavius* | Asama | Mole | WA**V**SA | 649-653 |
|  | Bruges | Mole | WA**V**SA | 649-653 |
|  | Fugong | Vole | WA**V**SA | 648-652 |
|  | Hantaan | Murine | WAASA | 644-648 |
|  | Oxbow | Mole | WAASA | 650_654 |
|  | Puumala | Vole | WAASA | 654-658 |
|  | Rockport | Mole | WAASA | 646-650 |
| *Thottimvirus* | Imjin | Shrew | WAASA | 632-636 |
|  | Thottapalayam | Shrew | WAASA | 633-637 |

**Table S5: Results of RT-qPCR analyses of different tissues of Brno virus-infected common noctule bats from Germany (DE) and nested RT-PCR results for bat samples from Poland (POL).**

| Organ | BH8/16-23/DE  (RT-qPCR) | BH8/16-276/DE  (RT-qPCR) | BH8/16-347/DE  (RT-qPCR) | 22/2016/POL  (nested RT-PCR) | 28/2018/POL  (nested RT-PCR) | 29/2018/POL  (nested RT-PCR) |
| --- | --- | --- | --- | --- | --- | --- |
| brain | negative | (+) | + | n.d. | n.d. | n.d. |
| lung | + | +++ | + | negative | negative | positive |
| liver | ++ | +++ | ++ | positive | negative | positive |
| kidney | + | + | + | negative | positive | negative |
| spleen | negative | negative | + | n.d. | n.d. | n.d. |
| intestine | (+) | + | negative | n.d. | n.d. | n.d. |
| body cavity fluid | negative | (+) | negative | n.d. | n.d. | n.d. |

cycle threshold (ct) values: ≥40, negative; ≥35 and <40, (+) questionable; ≥30 and <35, + low viral genome load; ≥25 and <30, ++ moderate viral genome load; ≥20 and <25, +++ high viral genome load; <20, ++++ very high viral genome load

n.d., not determined

**References**

1. Klempa B, Fichet-Calvet E, Lecompte E, Auste B, Aniskin V, Meisel H, Denys C, Koivogui L, ter Meulen J, Krüger DH (2006) Hantavirus in African wood mouse, Guinea. Emerg Infect Dis 12:838-840. <https://doi.org/10.3201/eid1205.051487>
