# Supplementary material for: Hantavirus Brno loanvirus is highly specific to the common noctule bat (Nyctalus noctula) and widespread in Central Europe
Source: Virus Genes. 2022 Dec 21;59(2):323–32. doi: 10.1007/s11262-022-01952-2 (PMC10025241; doi:10.1007/s11262-022-01952-2)
Supplement: Supplementary file 3 — Supplementary file3 (DOCX 39 kb) [file 11262_2022_1952_MOESM3_ESM.docx]

**Supplementary Information**

**Hantavirus Brno loanvirus is highly specific to the common noctule bat (*Nyctalus noctula*) and widespread in Central Europe**

Maysaa Dafalla^1^, Anna Orłowska^2^, Sinan Julian Keleş^3^, Petra Straková^4^, Kore Schlottau^5^, Kathrin Jeske^1^, Bernd Hoffmann^5^, Gudrun Wibbelt^6^, Marcin Smreczak^2^, Thomas Müller^7^, Conrad Martin Freuling^7^, Xuejing Wang^9^, Jerzy Rola^2^, Stephan Drewes^1^, Sasan Fereidouni^3^, Gerald Heckel^8, 9^, Rainer G. Ulrich^1,10,*^

^1^Friedrich-Loeffler-Institut, Federal Research Institute for Animal Health, Institute of Novel and Emerging Infectious Diseases, Südufer 10, 17493 Greifswald-Insel Riems, Germany, ^2^Department of Virology, National Veterinary Research Institute, 57 Partyzantów Avenue, 24-100 Pulawy, Poland, ^3^Research Institute of Wildlife Ecology, University of Veterinary Medicine Vienna, Savoyenstraße 1a,1160 Vienna, Austria, ^4^Veterinary Research Institute, Hudcova 296/70, 621 00 Brno, Czech Republic, ^5^Institute of Diagnostic Virology, Friedrich-Loeffler-Institut, Federal Research Institute for Animal Health, Südufer 10, 17493 Greifswald-Insel Riems, Germany, ^6^Leibniz-Institute for Zoo and Wildlife Research, Alfred-Kowalke-Straße 17, 10315 Berlin, Germany, ^7^Institute of Molecular Virology and Cell Biology, Friedrich-Loeffler-Institut, Federal Research Institute for Animal Health, Südufer 10, 17493 Greifswald-Insel Riems, Germany, ^8^University of Bern, Institute of Ecology and Evolution, Baltzerstrasse 6, 3012 Bern, Switzerland, ^9^Swiss Institute of Bioinformatics, Quartier Sorge - Batiment Amphipole, 1015 Lausanne, Switzerland, ^10^Deutsches Zentrum für Infektionsforschung (DZIF), Partner site Hamburg-Lübeck-Borstel-Riems, Südufer 10, 17493 Greifswald-Insel Riems, Germany

***Corresponding author:** [rainer.ulrich@fli.de](mailto:rainer.ulrich@fli.de)

**Collection of bats, dissection and test strategy**

For surveillance of bat rabies, bats were collected in 11 German federal states [[1](#_ENREF_1)], Poland and Austria – from which, samples were collected in the framework of bat rabies surveillance and bat virus infection monitoring studies. Animals from Germany and Poland were dissected following a standard protocol, and tissue samples (lung, liver, spleen, kidney, brain, intestine) and body cavity fluid were collected. In Austria, in addition to the dead bat carcasses, swab samples were collected from oro-pharyngeal and rectal cavities, and transferred into viral transport medium (phosphate buffer saline (PBS), containing antibiotics and 5% calf serum). Bat species identification was done morphologically and, for bat carcasses in Poland and Germany, using molecular methods [[1](#_ENREF_1), [2](#_ENREF_2)].

**Nucleic acid extraction, RT-PCR analysis and sequencing**

Pea-sized tissue samples from bats from Germany and Austria were homogenized in 500µl PBS and a pool, consisting of all six organs from each animal, was generated for RT-qPCR screening. Pool RNA was extracted using Microlab Star automate (Hamilton Robotics, Reno, NV, USA) in combination with the Nucleo Spin 96 Virus Core Kit (Macherey-Nagel, Düren, Germany) according to the manufacturer’s instructions. In case of positive animals, all organs were subsequently extracted individually for further analysis.

RNA of each animal was subjected to bat hantavirus RNA screening using Brno loanvirus (BRNV) specific RT-qPCR [[3](#_ENREF_3)]. This RT-qPCR targets the L segment and results in a product of 109 base pairs that overlaps the product of the conventional nested RT-PCR (for primer sequences and positions see Table S1). In addition, lung sample-derived RNAs of all common noctules were reverse transcribed into cDNA using SuperScriptIII One-step RT-PCR Kit (Thermo Fisher Scientific, (Invitrogen, Darmstadt, Germany) and cDNA was amplified using a previously published generic L segment conventional PCR [[4](#_ENREF_4)]. Further, almost complete coding S and M segment sequences were determined using primers given in Table S1. The amplification of specific products was resolved by agarose gel electrophoresis. DNA sequence determination was done with the BigDye Terminator v1.1 cycle Sequencing Kit (Thermo Fisher Scientific) on a 3130 Genetic Analyzer (Life Technologies, Darmstadt, Germany).

For bat samples from Poland, RNA was isolated separately from 10% lung, liver and kidney homogenates (w/v). Subsequently, pools consisting of all three RNA preparations for each animal were used in nested RT-PCR, following a previously published protocol [[4](#_ENREF_4)]. RT-PCR was performed using SuperScriptIII One-step RT-PCR Kit (Thermo Fisher Scientific), whereas for the nested PCR, Hot-star Taq DNA polymerase (Qiagen, Hilden, Germany) was applied. All hantavirus positive pools were subjected to individual testing, i.e. hantavirus RNA detection in each lung, kidney and liver of individual bats. Specific amplicons were detected by agarose gel electrophoresis and then sequenced on an automated sequencer ABI PRISM 310 Genetic Analyzer (Applied Biosystems, Waltham, MA, USA) using a BigDye Sequencing Kit (Applied Biosystems) with GeneScan Analysis Software.

**Sequence comparison and phylogenetic analyses**

Alignments of partial L and (almost) complete S and M segment sequences were constructed using the Clustal W Multiple Alignment algorithm implemented in Bioedit v7.2.3. [[5](#_ENREF_5)]. The most suitable substitution model was determined by jModelTest v2.1.6 [[6](#_ENREF_6)]. The consensus trees are based on Bayesian analyses with 6 x 10^6^ to 1 x 10^7^ generations and a burn-in phase of 25% using MrBayes v3.2.6 [[7](#_ENREF_7)], and Maximum-Likelihood analyses with 1,000 bootstrap replicates performed with FastTreeMP v2.1.10 [[8](#_ENREF_8)] and 50% cut-off using the General Time Reversible (GTR) substitution model with invariant sites and a gamma distributed shape parameter for both algorithms. The consensus tree was established by transferring the bootstrap values to the Bayesian tree only if branches were supported by both trees. All tree reconstructions were done on CIPRES Science Gateway [[9](#_ENREF_9)].

**Functional protein domain prediction and sliding window analysis**

The complete coding S and M segment sequences from BRNV protype strain Brno 7_2012 (accession numbers: KX845678 and KX845679) were translated to protein sequences using BioEdit (V7.2.3.) [[5](#_ENREF_5)]. For functional protein domain analysis, the InterPro Classification of protein families from the European Molecular Biology Laboratory – European´s Bioinformatic Institute (EMBL-EBI) (<https://www.ebi.ac.uk/interpro/>) was used and results are shown schematically.

Amino acid diversity along each protein for each clade, as well as the amino acid divergence between clade I and clade IIa or clade IIb, was calculated using a sliding window method. To avoid potential bias caused by unequal sample sizes of sequences, seven viruses were selected to represent each clade (see Fig. S1). Pairwise differences of amino acids between the selected sequences were calculated using SSE v1.4 [[10](#_ENREF_10)] with a window length of 40 amino acids and a step size of 20 amino acids. The average numbers of amino acid differences were then calculated in R [[11](#_ENREF_11)].

**References**

1. Schlottau K, Eggerbauer E, Freuling CM, Beer M, Müller T, Hoffmann B (2020) Rapid molecular species identification of indigenous bats from Germany for surveillance purposes. Infect Genet Evol 78:104140. <https://doi.org/10.1016/j.meegid.2019.104140>

2. Harris SL, Brookes SM, Jones G, Hutson AM, Racey PA, Aegerter J, Smith GC, McElhinney LM, Fooks AR (2006) European bat lyssaviruses: Distribution, prevalence and implications for conservation. Biol Conserv 131:193-210. <https://doi.org/10.1016/j.biocon.2006.04.006>

3. Straková P, Dufkova L, Širmarová J, Salát J, Bartonička T, Klempa B, Pfaff F, Höper D, Hoffmann B, Ulrich RG, Růžek D (2017) Novel hantavirus identified in European bat species *Nyctalus noctula*. Infect Genet Evol 48:127-130. <https://doi.org/10.1016/j.meegid.2016.12.025>

4. Klempa B, Fichet-Calvet E, Lecompte E, Auste B, Aniskin V, Meisel H, Denys C, Koivogui L, ter Meulen J, Krüger DH (2006) Hantavirus in African wood mouse, Guinea. Emerg Infect Dis 12:838-840. <https://doi.org/10.3201/eid1205.051487>

5. Hall TA (1999) BioEdit: a user-friendly biological sequence alignment editor and analysis program for Windows 95/98/NT. Nucl Acids Symp Ser 41:95-98.

6. Darriba D, Taboada GL, Doallo R, Posada D (2012) jModelTest 2: more models, new heuristics and parallel computing. Nat Methods 9:772. <https://doi.org/10.1038/nmeth.2109>

7. Ronquist F, Teslenko M, van der Mark P, Ayres DL, Darling A, Höhna S, Larget B, Liu L, Suchard MA, Huelsenbeck JP (2012) MrBayes 3.2: efficient Bayesian phylogenetic inference and model choice across a large model space. Syst Biol 61:539-542. <https://doi.org/10.1093/sysbio/sys029>

8. Price MN, Dehal PS, Arkin AP (2010) FastTree 2 - approximately maximum-likelihood trees for large alignments. PLoS One 5:e9490. <https://doi.org/10.1371/journal.pone.0009490>

9. Miller MA, Pfeiffer W, Schwartz T (2010) Creating the CIPRES Science Gateway for inference of large phylogenetic trees. 2010 gateway computing environments workshop (GCE). IEEE, New Orleans, LA, USA, pp. 1-8. <https://doi.org/10.1109/GCE.2010.5676129>

10. Simmonds P (2012) SSE: a nucleotide and amino acid sequence analysis platform. BMC Res Notes 5:50. <https://doi.org/10.1186/1756-0500-5-50>

11. R Core Team (2018) R: A language and environment for statistical computing. R Foundation for Statistical Computing, Vienna, Austria. Available online at: <https://www.R-project.org/>
